# The mouse claustrum synaptically connects cortical network motifs

**DOI:** 10.1016/j.celrep.2022.111860

**Published:** 2022-12-20

**Authors:** Houman Qadir, Brent W. Stewart, Jonathan W. VanRyzin, Qiong Wu, Shuo Chen, David A. Seminowicz, Brian N. Mathur

**Affiliations:** 1 Department of Pharmacology, University of Maryland School of Medicine, HSF III 9179, Baltimore, MD 21201, USA; 2 Department of Neural and Pain Sciences, University of Maryland School of Dentistry, Baltimore, MD, USA; 3 Division of Biostatistics and Bioinformatics, Department of Epidemiology & Public Health, University of Maryland School of Medicine, Baltimore, MD, USA; 4 Department of Biostatistics, Epidemiology and Informatics, University of Pennsylvania, Philadelphia, PA, USA; 5 Department of Medical Biophysics, Schulich School of Medicine & Dentistry, University of Western Ontario, London, ON, Canada; 6 Lead contact

## Abstract

Spatially distant areas of the cerebral cortex coordinate their activity into networks that are integral to cognitive processing. A common structural motif of cortical networks is co-activation of frontal and posterior cortical regions. The neural circuit mechanisms underlying such widespread inter-areal cortical coordination are unclear. Using a discovery based functional magnetic resonance imaging (fMRI) approach in mouse, we observe frontal and posterior cortical regions that demonstrate significant functional connectivity with the subcortical nucleus, the claustrum. Examining whether the claustrum synaptically supports such frontoposterior cortical network architecture, we observe cortico-claustro-cortical circuits reflecting the fMRI data: significant *trans*-claustral synaptic connectivity from frontal cortices to posteriorly lying sensory and sensory association cortices contralaterally. These data reveal discrete cortical pathways through the claustrum that are positioned to support cortical network motifs central to cognitive control functions and add to the canon of major extended cortico-subcortico-cortical systems in the mammalian brain.

## In brief

Cortical networks are composed of geographically distinct frontal and posterior cortices. Qadir et al. show that the claustrum relays frontal cortical input to contralateral frontal and posterior cortices in a source-, cell-type-, and target-specific manner to support cortical network architecture.

## Graphical Abstract

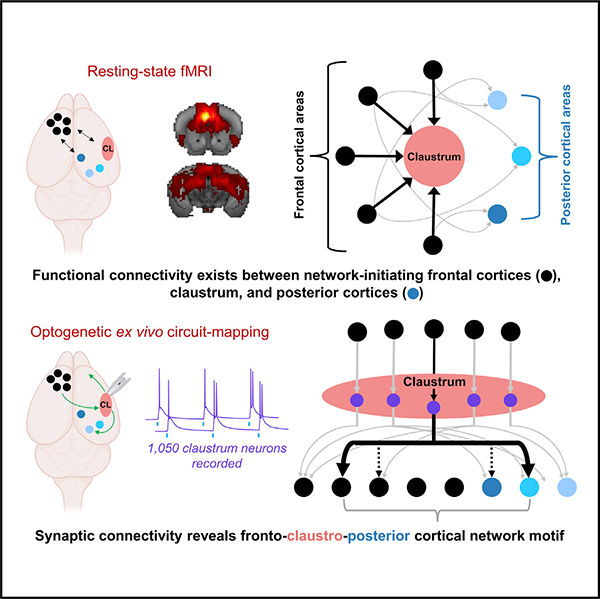

## INTRODUCTION

The transfer of executive cortical information through subcortical structures that lead back to the cortex is essential for cognition and the implementation of complex behavioral strategies.^1–7^ Such classical extended cortical systems include cortico-basal ganglia-cortical and cortico-thalamo-cortical loops. Delineating the specific directionality of flow of information through these multi-synaptic pathways (e.g., through direct and indirect pathways of the basal ganglia) has proven critical to advancing our understanding of their functional attributes.^8–11^ How any of these extended cortical systems give rise to synchronized inter-areal cortical activity states, known as cortical networks, that critically support cognition is unclear.

Data suggest that cortical networks may be initiated by frontal cortical regions,^12^ and cognitive control processes may originate in frontal cortices.^13–15^ An understudied, yet significant, projection system emanating largely from frontal cortices routes to the subcortical nucleus is the claustrum. The claustrum^16,17^ and its frontal cortical input^18^ are required for optimal performance during cognitively demanding tasks in mice. In humans, the claustrum is activated during execution of difficult, not easy, versions of the multi-source interference attention task; this activation occurs coincidently with the emergence of task-positive cortical networks.^19^ Both task-positive networks and default mode network cortical areas are functionally connected with the claustrum at rest.^19,20^ Thus, the claustrum is positioned as a subcortical structure that may support cortical networks through discrete frontal cortico-claustro-cortical pathways.

While evidence exists supporting claustrum functional connectivity with the salience network in rat,^21^ and a degree of synaptic connectivity supports this,^22^ further investigation of how the claustrum may provide a circuit mechanism supporting cortical network motifs composed of frontal and posterior cortical regions, such as task-positive and default mode networks, is lacking. Elucidating a circuit mechanism supporting network communication may provide critical insight to myriad neuropsychiatric disorders in which the loss of network integrity predicts cognitive dysfunction, including addiction,^23^ depression,^24^ and schizophrenia.^25,26^

To address this, we analyzed the resting-state functional connectivity (rsFC) of the five anatomically and functionally well-characterized mouse frontal cortical areas using functional magnetic resonance imaging (fMRI) data to assess claustrum functional connectivity. Testing the possible structural and synaptic connectivity underlying the functional connectivity of regions of interest identified using this approach, we examined 35 unique frontal cortico-claustral-cortical circuits using synaptic circuit mapping across 1,050 claustrum projection neurons of two physiologically distinct subtypes.^27^ These data reveal distinct, primary information pathways through the claustrum that reflect a motif common in cortical networks underlying cognition.

## RESULTS

### Mouse fMRI reveals rsFC between frontal cortical regions and claustrum

**Figure 1.**
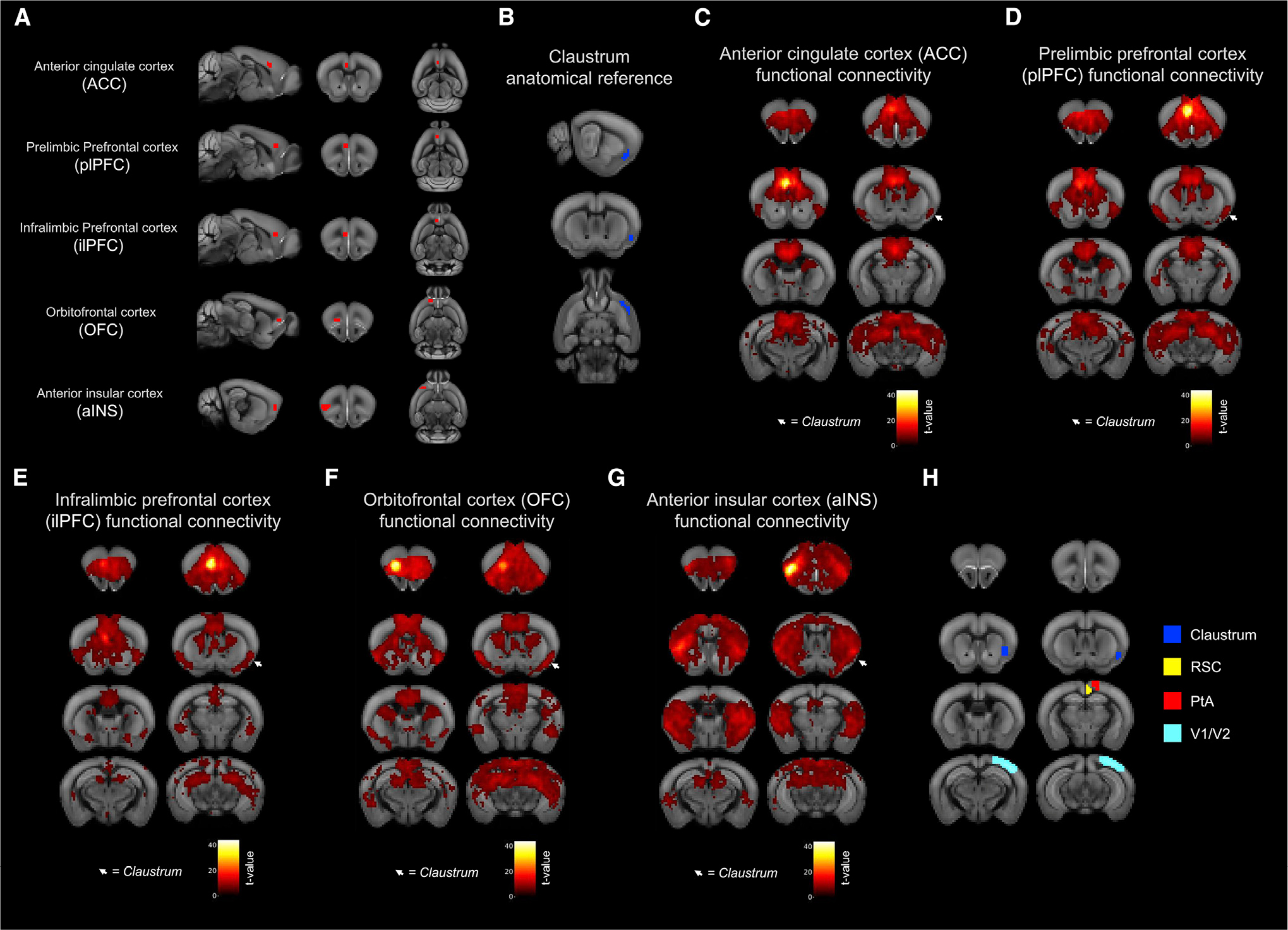
Frontal cortex seeds exhibit resting-state functional connectivity (rsFC) with the claustrum (A) Sagittal (left), coronal (middle), and axial (right) views of unilateral anterior cingulate cortex (ACC); prelimbic prefrontal cortex (plPFC); infralimbic prefrontal cortex (ilPFC); orbitofrontal cortex (OFC); and anterior insular cortex (aINS) seed regions of interest (ROIs) as drawn overlaid on the Allen Institute for Brain Science (AIBS) mouse template. (B) Coronal slices displaying the rostro-caudal extent of the contralateral claustrum ROI overlaid on the AIBS mouse template. (C–G) Slices correspond to those over which the rsFC heatmaps of (C) ACC, (D) plPFC, (E) ilPFC, (F) OFC, and (G) aINS are displayed (multiple comparisons-corrected voxel-wise FWE p < 0.05). Color bars indicate t-statistic values. White arrows indicate regions of rsFC overlap with contralateral claustrum. (H) Coronal slices displaying claustrum with parietal cortices including retrosplenial cortex (RSC), parietal association cortex (PtA), and visual cortex (V1/V2).

Both task-positive and default mode networks are composed of specific frontal and posterior cortical regions.^28–31^ The default mode network includes the ventromedial prefrontal cortex and the posterior cingulate cortex.^32,33^ Previous imaging data reveal that such anti-correlated networks are conserved, to an extent, across rodents.^34,35^ Since cortical networks are initiated by their frontal cortical components,^12^ we sought to examine the functional connectivity of a host of well-characterized frontal regions in mice including the anterior cingulate cortex (ACC); prelimbic prefrontal cortex (plPFC); infralimbic prefrontal cortex (ilPFC); the orbitofrontal cortex (OFC); and anterior insular cortex (aINS). To assess whether these cortical areas possess functional connectivity with the claustrum, we used a publicly available fMRI dataset (Donders Repository: https://public.data.donders.ru.nl/dcmn/DSC_4180000.18_502_v1) acquired at 9.4 T (n = 51 mice). Following selection of unilateral (left) regions of interest (ROIs) for all five frontal seed regions (Figure 1A), rsFC maps for each frontal cortex seed exhibited significantly connected voxels surviving a conservative voxel-wise correction for multiple comparisons (family-wise error [FWE] p < 0.05) within bilateral claustrum (CL) regions (Figures 1B–1G). In addition, significantly connected voxels were also observed in posterior cortical regions, including the retrosplenial cortex (RSC), parietal association cortex (PtA), and visual cortex (V1/V2) (Figure 1H).

### Claustrum projection neuron subtypes differ by firing properties and morphology

Functional connectivity analyses reveal voxels with timeseries significantly correlated with a seed region, and functional connectivity often reflects anatomical features.^36,37^ However, functional and anatomical connectivity do not necessarily correspond with, nor can they be definitively interpreted as, evidence of a direct influence of one brain region on another.^38^ Consequently, the rsFC data, while suggestive, do not allow conclusions regarding underlying synaptic connections nor cellular subtype specificity. We therefore investigated the strength of synaptic connectivity in distinct cortico-claustro-cortical circuits originating from the five frontal cortex seed regions chosen for rsFC analysis above and terminating either ipsilaterally back to the originating frontal cortical regions or to the posterior cortical network regions identified in our rsFC data maps (Figure 1H).

**Figure 2.**
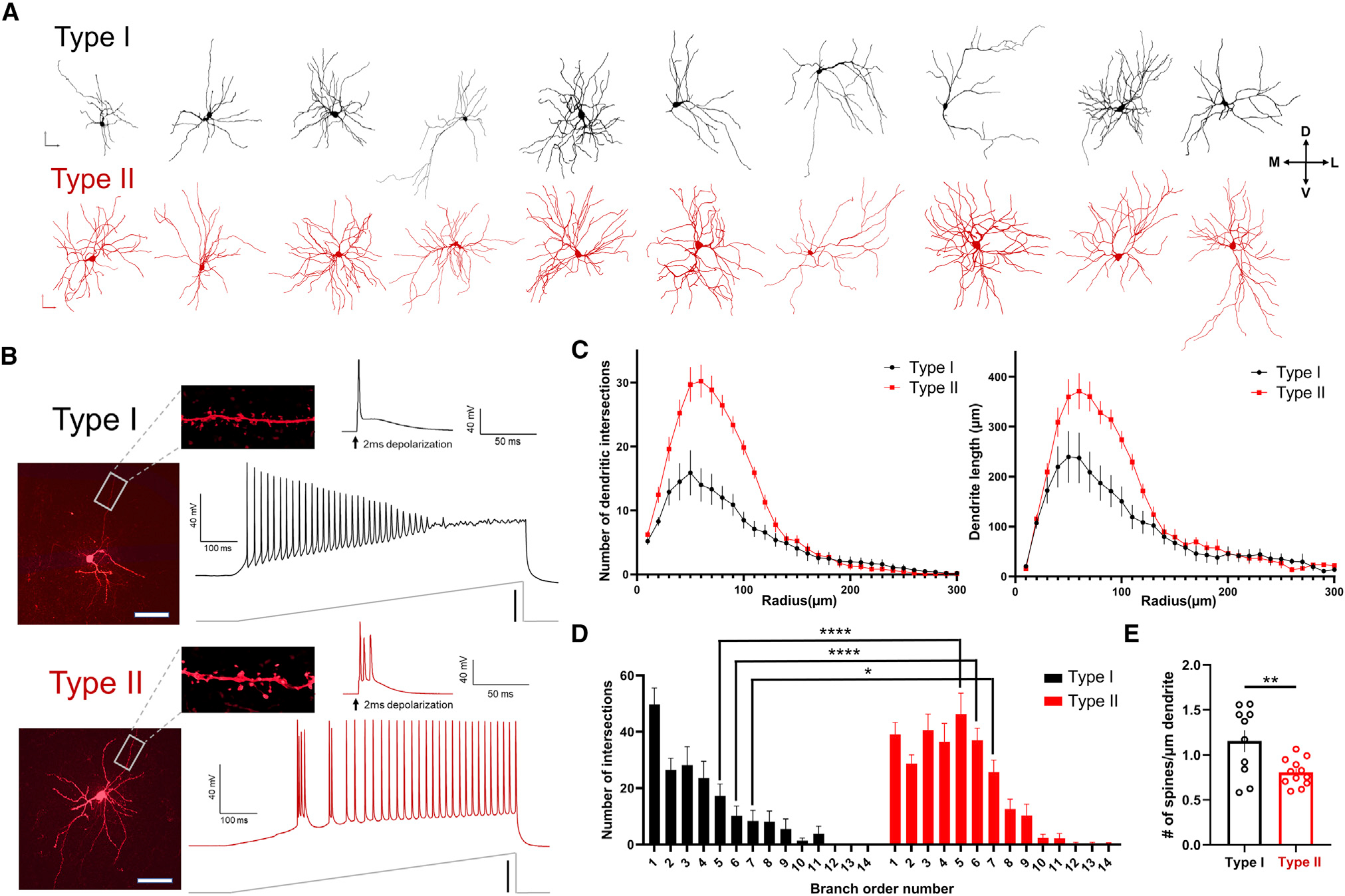
Claustrum type II spiny projection neurons dendritic morphology is more complex than that of type I neurons (A) Top: representative cell fill 3D reconstructions of type I claustrum projection neurons. Bottom: representative three-dimensional reconstructions of type II claustrum projection neurons (n = 10 cells shown for each subtype). (B) Top: type I claustrum projection neurons were defined by a lack of burst firing following a 2 ms depolarization voltage step. Representative voltage trace following a current-injection ramp. Bottom: type II claustrum projection neurons were defined by the presence of burst firing following a 2 ms depolarization voltage step. Shown is a representative voltage trace following a current-injection ramp. (C) Type II claustrum neurons displayed a greater number of dendritic intersections and increased dendrite length compared with type I neurons (Kruskal-Wallis test: p < 0.0001; type I: n = 11 cells; type II: n = 12 cells). (D) Type II neurons displayed an increased number of intersections of higher branch order numbers (two-way ANOVA: F(13,308) = 3.97, p < 0.0001, Bonferroni post hoc: p < 0.01). (E) Type I neurons displayed an increased number of spines per μm of dendrite (unpaired t test, p = 0.008). Vertical scale bars: (A) 100 μm; (B) current ramp: 200 pA. Horizontal scale bars: (A) 100 μm and (B) 200 μm.

Previous work suggests the existence of two potential projection neuron subtypes in the claustrum that could support cortico-claustro-cortical circuits.^27^ To extend known differences between these subtypes, we sought to determine whether morphological features map onto known electrophysiological differences. To do this, mice of both sexes received bilateral injections of an anterograde GFP-expressing virus (AAV5-hSyn-EGFP) in the ACC. This resulted in a fluorescent ACC axon terminal field in the claustrum that is isomorphic with immunostaining for the claustrum marker parvalbumin.^18,39^ We recorded from claustrum projection neurons using a biocytin-filled internal recording solution to create three-dimensional reconstructions of the recorded neurons (Figure 2A). The identification of each claustrum projection neuron was determined based on burst-firing properties (Figure 2B): “type II” claustrum projection neurons burst fire (defined as more than one action potential) following a brief 2 ms depolarizing voltage step, whereas “type I″ neurons do not (i.e., they fire single action potentials).^27^ Following Sholl analysis of the reconstructed claustrum neurons, type II neurons exhibited a significantly greater dendritic length and number of dendritic intersections than type I neurons (Figure 2C). The increased number of intersections appeared in higher branch order numbers (Figure 2D). While type II gross dendritic morphology was more complex than that of type I neurons, type I neurons exhibited greater dendritic spine density compared with type II neurons (Figure 2E). Since both projection neuron subtypes significantly differed on physiological and morphological grounds, we heretofore tested each neuronal subtype for differences in cortico-claustro-cortical connectivity.

### Structural connectivity suggests multiple frontal cortico-claustral-cortical circuits exist

**Figure 3.**
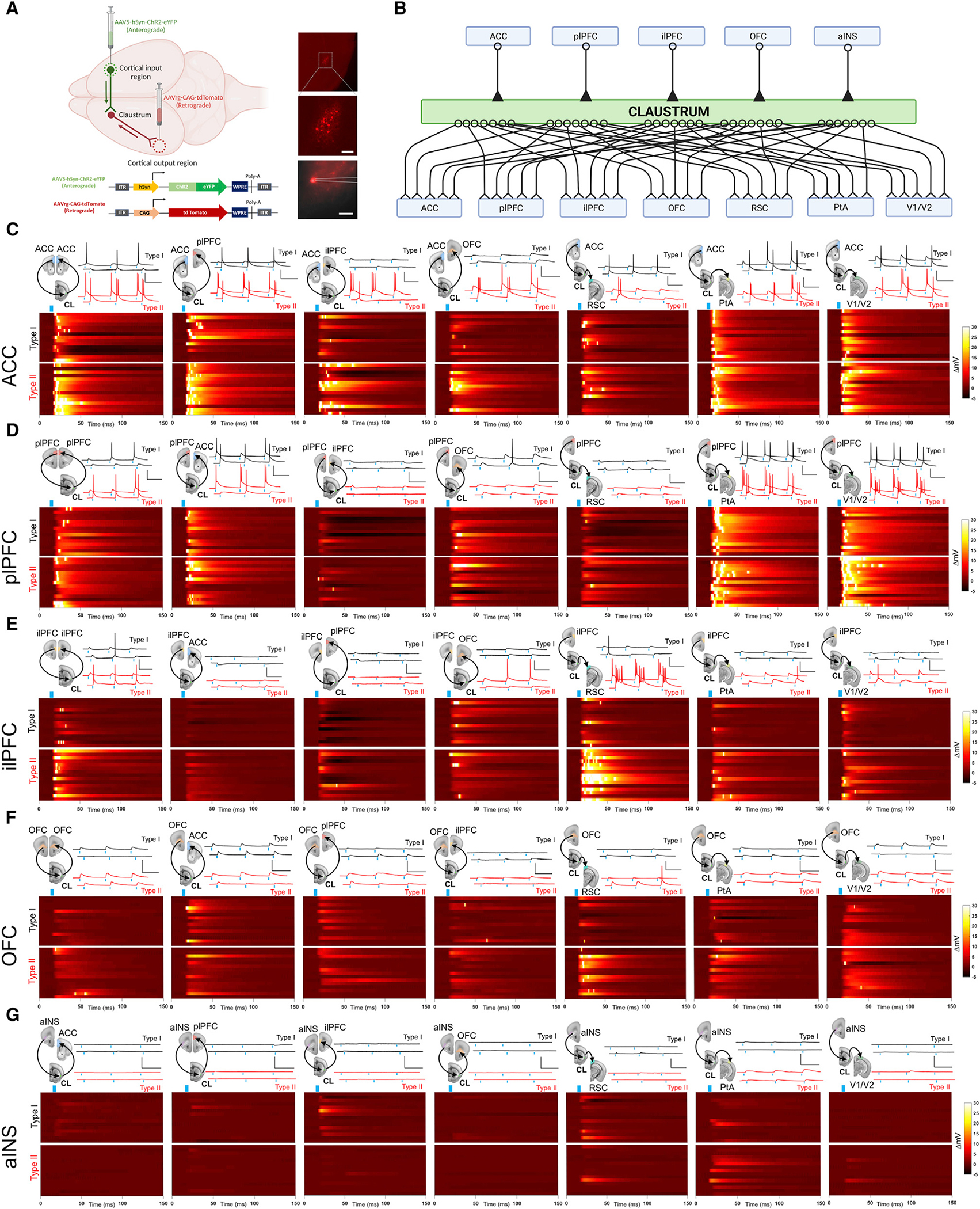
Functional channelrhodopsin-assisted circuit mapping of frontal cortico-claustro-cortical circuits (A) Left: illustration of viral setup for the structural circuit mapping method. AAV5-hSyn-ChR2-EYFP was injected into the input cortical nucleus for anterograde terminal labeling and AAVrg-CAG-tdTomato was injected in output cortical nuclei for retrograde soma labeling in claustrum. Right: representative image of fluorescently labeled spiny claustrum projection neurons for fluorescence-guided slice recordings. (B) Cartoon of all 35 frontal cortical-claustro-cortical circuits tested with 5 input frontal cortical regions and 7 output regions. (C) Top: diagram of *ex vivo* ACC *trans*-claustral circuits tested (projecting through claustrum to ACC, plPFC, ilPFC, OFC, RSC, PtA, and V1/V2 cortices; n = 15 type I cells; n = 15 type II cells each circuit) and corresponding representative voltage traces for recorded type I and II claustrum neurons. Bottom: heatmaps depicting average change in membrane potential across each recording following each light pulse stimulation (blue marker) for type I and II claustrum neurons. Only recordings from maximum light intensity (3 mW) are shown. (D) Diagram of *ex vivo* plPFC *trans*-claustral circuits tested (projecting to plPFC, ACC, ilPFC, OFC, RSC, PtA, and V1/V2 cortices, respectively; n = 15 type I cells; n = 15 type II cells each circuit) and corresponding representative voltage traces for recorded type I and II claustrum neurons. (E) Diagram of *ex vivo* ilPFC *trans*-claustral circuits tested (projecting to ilPFC, ACC, plPFC, OFC, RSC, PtA, and V1/V2 cortices; n = 15 type I cells; n = 15 type II cells each circuit) and corresponding representative voltage traces for recorded type I and II claustrum neurons. (F) Diagram of *ex vivo* OFC *trans*-claustral circuits tested (projecting to OFC, ACC, plPFC, ilPFC, RSC, PtA, and V1/V2 cortices; n = 15 type I cells; n = 15 type II cells each circuit) and corresponding representative voltage traces for recorded type I and II claustrum neurons. (G) Diagram of *ex vivo* aINS *trans*-claustral circuits tested (projecting to ACC, plPFC, ilPFC, OFC, RSC, PtA, and V1/V2 cortices, respectively; n = 15 type I cells; n = 15 type II cells each circuit) and corresponding representative voltage traces for recorded type I and II claustrum neurons. n = 1,050 cells total. Horizontal scale bars: (A) top: 500 μm, bottom: 200 μm and (C–G) 100 ms. Vertical scale bars: (C–G) 40 mV. Portions of this figure were created with BioRender.com.

We next endeavored to test the 35 possible frontal cortico-claustro-cortical circuits emanating from our five frontal cortical fMRI ROIs through both claustrum projection neuron subtypes and out to (1) the originating frontal regions and (2) the posterior cortical regions identified in the rsFC data (Figures 3A and S1A). We bilaterally injected an anterograde EYFP virus (AAV5-hSyn-ChR2-EYFP) into various frontal cortical regions (Figure 3B) including the ACC (Figure S1B); plPFC (Figure S1C); ilPFC (Figure S1D); OFC (Figure S1E); and aINS (Figure S1F). A retrograde tdTomato virus (AAVrg-CAG-tdTomato) was also injected bilaterally in either the ACC (Figure S1G); plPFC (Figure S1H); ilPFC (Figure S1I); OFC (Figure S1J); aINS (Figure S1K); PtA (Figure S1L); V1/V2 (Figure S1M); and RSC (Figure S1N) to observe overlap between anterograde and retrograde labeling within the claustrum. We observed dense terminal anterograde expression throughout the rostral-caudal axis of the claustrum from the ACC (Figure S1B), plPFC (Figure S1C), and ilPFC (Figure S1D). Moderate EYFP expression was observed in the claustrum following bilateral injections in the OFC (Figure S1E), and sparse labeling was observed from the aINS in the claustrum (Figure S1F). Anterograde axon terminal expression from all frontal regions tested, except aINS, was localized within the claustrum with little to no expression in the surrounding insular cortex or dorsal endopiriform nucleus (Figures S1A–S1F). Inputs arising from parietal sensory regions were not tested since networks are initiated by frontal cortical regions^12^ and optogenetic stimulation of sensory cortical inputs to claustrum fail to elicit significant neuronal depolarization in claustrum projection neurons.^18^ We found that fluorescent retrogradely labeled cell bodies in the claustrum targeted the ACC (Figure S1G); plPFC (Figure S1H); ilPFC (Figure S1I); OFC (Figure S1J); PtA (Figure S1L); V1/V2 (Figure S1M); and RSC (Figure S1N). We found no cells labeled in the claustrum following retrograde viral injection in the aINS (Figure S1K), which confirms a previous report.^40^

### Synaptic circuit mapping reveals distinct cortico-claustro-cortical circuits

To determine whether the structural connections observed indeed form synaptically connected cortico-claustro-cortical pathways—and to what degree of strength they form—we used a channelrhodopsin 2 (ChR2)-assisted circuit mapping approach. We injected anterogradely transported AAV5-hSyn-ChR2-EYFP in each of the frontal cortical seed regions used previously (Figures 1 and 3): ACC, plPFC, ilPFC, OFC, and aINS. Retrogradely labeled tdTomato-positive claustrum projection neurons were recorded using whole-cell patch-clamp electrophysiology for each cortico-claustro-cortical circuit (Figure 3A) while stimulating presynaptic axon terminals with varying blue light intensities (Figure S2). Each neuron was first categorized as a type I or II claustrum projection neuron based on the action potential firing response to a brief depolarizing voltage step (Figure 2B), as previously reported.^27^

**Figure 4.**
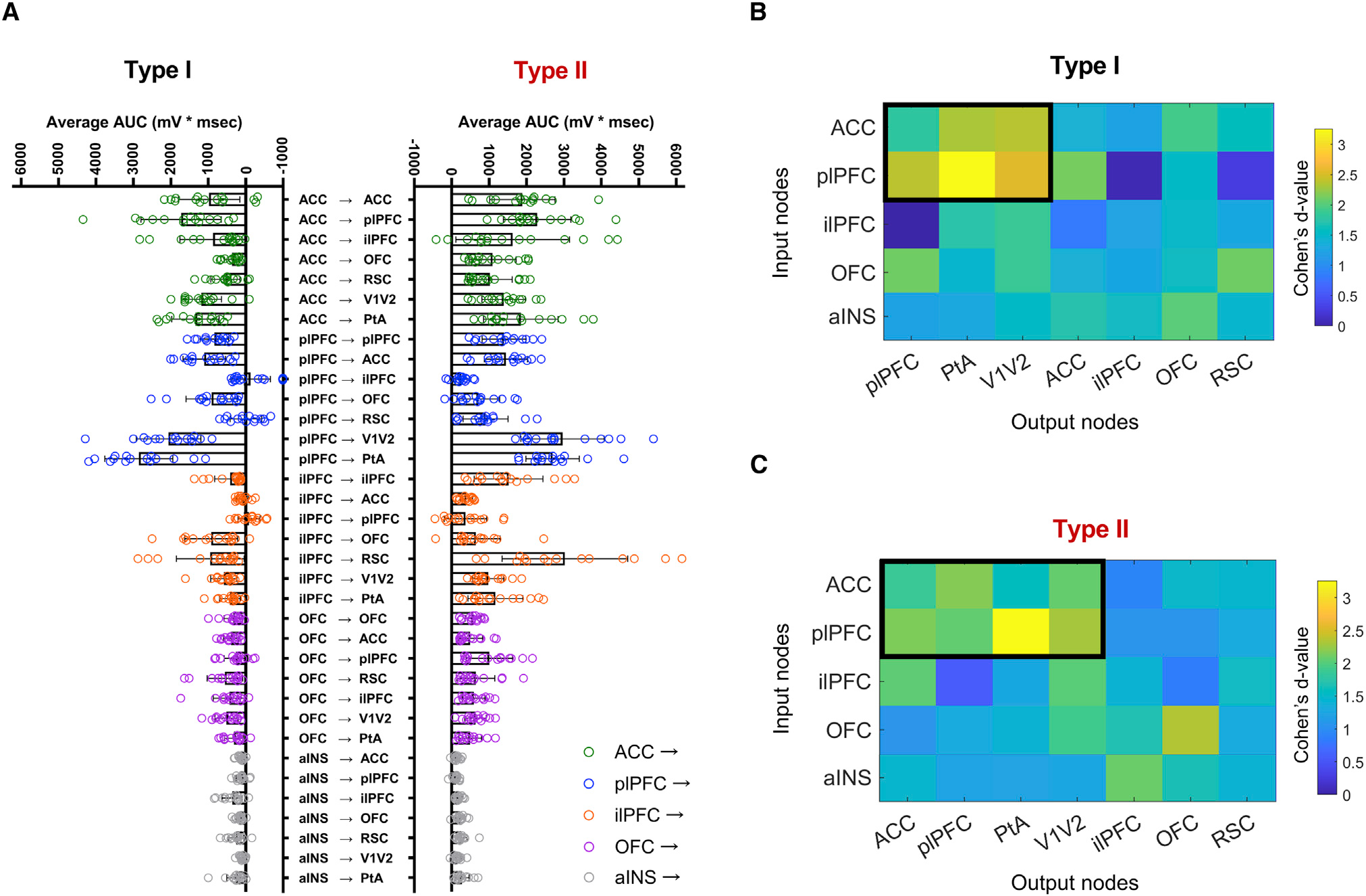
Average area under the curve metric shows specificity in circuit strength based on claustrum neuron output target region and claustrum projection neuron subtype (A) Averaging the area under the curve (AUC) of each voltage trace (for all light intensities) reveals differences in circuit strength across frontal cortical *trans*-claustral circuits depending on type I and II claustrum projection neuron target (Kruskal Wallis test for multiple comparisons: p < 0.0001; n = 525 type I cells and n = 525 type II cells total). (B and C) Clustered frontal cortico-claustro-cortical circuits were detected by subgraph extraction (cluster marked in black) for type I and type II claustrum neuron subtypes. Type I: detected subgraph from ACC and plPFC to plPFC, PtA, and V1/V2. Type II: detected subgraph from ACC and plPFC to ACC, plPFC, PtA, and V1/V2. Detected subgraphs p < 0.001 under a permutation test. Error bars: standard error of the mean.

**Figure 5.**
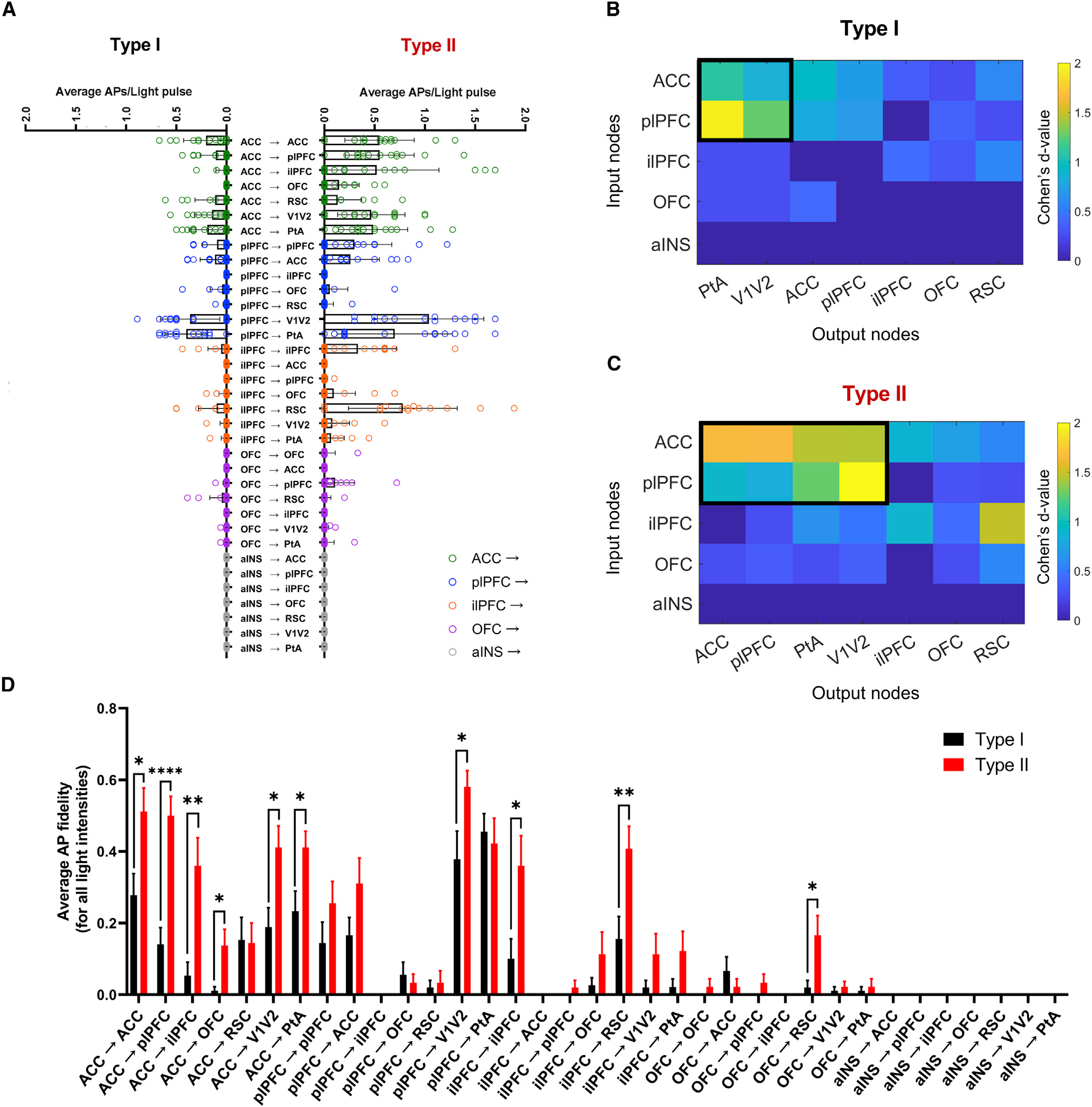
Average action potentials per light pulse metric shows specificity in circuit strength based on claustrum neuron output target region and claustrum projection neuron subtype (A) Average action potentials (APs) per light pulse stimulation of each voltage trace (for all light intensities) revealed differences in circuit strength across frontal cortical *trans*-claustral circuits depending on type I and II claustrum projection neuron target (Kruskal Wallis test for multiple comparisons: p < 0.0001; n = 570 type I cells and n = 525 type II cells total). (B and C) Clustered frontal cortico-claustro-cortical circuits were detected by subgraph extraction (cluster marked in black) for type I and type II claustrum neuron subtypes. Type I: detected subgraph from ACC and plPFC to PtA and V1/V2. Type II: detected subgraph from ACC and plPFC to ACC, plPFC, PtA, and V1/V2. Detected subgraphs p < 0.001 under permutation test. (D) Select frontal cortico-claustro-cortical circuits preferentially activated type II neurons compared with type I neurons: ACC > CL > ACC (Wilcoxon rank-sum test: p = 0.027); ACC > CL > plPFC (p < 0.0001); ACC > CL > ilPFC (p = 0.003); ACC > CL > OFC (p = 0.006); ACC > CL > V1/V2 (p = 0.016); ACC > CL > PtA (p = 0.009); plPFC > CL > V1/V2 (p = 0.041); ilPFC > CL > ilPFC (p = 0.016); ilPFC > CL > V1/V2 (p = 0.215); and OFC > CL > RSC (p = 0.015). n = 15 cells in each circuit for each subtype. n = 1,050 cells total. Error bars: standard error of the mean.

Based on area under the postsynaptic voltage response curve (AUC) (Table 1; Figure 4) and action potentials (APs) per light pulse synaptic strength metrics (Table 1; Figure 5), we discovered four frontal cortico-claustro-cortical circuits that terminated back on the originating cortical area on the contralateral side (e.g., left ACC > CL > right ACC) that we termed “homoloquial” (homo meaning same, and loquial meaning communicating) circuits (for lack of an existing term). These circuits include ACC > CL > ACC (Figures 3C, 4A, and 5A); plPFC > CL > plPFC (Figures 3D, 4A, and 5A); ilPFC > CL > ilPFC (Figures 3E, 4A, and 5A); and OFC > CL > OFC (Figures 3F, 4A, and 5A). An aINS homoloquial circuit was not tested as we found no claustrum neurons projected to the aINS (Figure S1K).

The other 31 circuits tested are circuits where inputs originate in frontal cortices and synapse onto claustrum neurons that project ipsilaterally to a different cortical area from the originating frontal cortical region termed “heteroloquial” circuits (hetero meaning different, and loquial meaning communicating). ACC-originating heteroloquial circuits (Figures 3C, 4A, and 5A) included ACC > CL > plPFC; ACC > CL > ilPFC; ACC > CL > OFC; ACC > CL > RSC; ACC > CL > PtA; and ACC > CL > V1/V2. Upon ACC input activation, we found that type I and II claustrum neurons projecting to all output areas displayed AP firing. However, the strongest postsynaptic responses were observed in plPFC-, PtA-, and V1/V2-projecting neurons (Figures 4 and 5; Table 1).

The plPFC-originating heteroloquial circuits (Figures 3D, 4A, and 5A) included plPFC > CL > ACC; plPFC > CL > ilPFC; plPFC > CL > OFC; plPFC > CL > RSC; plPFC > CL > PtA; and plPFC > CL > V1/V2. Notably, activation of plPFC inputs onto both type I and II claustrum neurons targeting PtA and V1/V2 resulted in the strongest postsynaptic responses out of all plPFC input circuits tested (Figures 4 and 5; Table 1). Inversely, activation of plPFC inputs onto largely type I claustrum neurons targeting ilPFC and RSC exhibited hyperpolarizing postsynaptic responses (Figures 4 and 5; Table 1). Hyperpolarizing responses were likely due to local polysynaptic inhibition as the GABA reversal potential for both type I and II neurons was hyperpolarized relative to the average resting membrane potential^27^ (Figure S3).

The ilPFC-originating heteroloquial circuits (Figures 3E, 4A, and 5A) included ilPFC > CL > ACC; ilPFC > CL > plPFC; ilPFC > CL > OF C; ilPFC > CL > RSC; ilPFC > CL > PtA; and ilPFC > CL > V1/V2. Notably, activation of ilPFC inputs onto type II RSC-projecting claustrum neurons resulted in the strongest postsynaptic responses (Table 1), whereas ilPFC input stimulation of plPFC-projecting neurons resulted in some hyperpolarizing responses.

The OFC-originating heteroloquial circuits (Figures 3F, 4A, and 5A) included OFC > CL > ACC; OFC > CL > plPFC; OFC > CL > ilPFC; OFC > CL > RSC; OFC > CL > PtA; and OFC > CL > V1/V2. Activation of OFC inputs onto all claustrum neurons generally resulted in postsynaptic depolarization events that did not achieve AP threshold.

Lastly, the aINS-originating heteroloquial circuits (Figures 3G, 4A, and 5A) included aINS > CL > ACC; aINS > CL > plPFC; aINS > CL > ilPFC; aINS > CL > OFC; aINS > CL > RSC; aINS > CL > PtA; and aINS > CL > V1/V2. Activation of aINS inputs failed to elicit observable postsynaptic responses for any recorded claustrum projection neuron.

It is important to note that while both male and female mice were used for all tested circuits, we did not observe any sex differences in our synaptic connectivity analyses (Figure S4).

### Cortico-claustral synaptic strength depends on claustrum neuron subtype and postsynaptic target

We next applied a subgraph extraction-based cluster analysis to determine whether any group of circuits in the electrophysiological dataset emerged as statistically significant patterns. By applying a permutation test (controlling for the FWE rate) with a significant p value set at ≤0.001, we detected a subgraph of circuits that originated in the ACC and plPFC for both type I (Figures 4B and 5B) and type II (Figures 4C and 5C) neurons. Based on the average AUC metric, the statistically significant clustered type I circuits included ACC > CL > plPFC (Cohen’s D value = 1.66); ACC > CL > PtA (2.23); ACC > CL > V1/V2 (2.28); plPFC > CL > plPFC (2.29); plPFC > CL > PtA (3.25); and plPFC > CL > V1/V2 (2.48). AUC metric clustered type II circuits included ACC > CL > ACC (2.24); ACC > CL > plPFC (2.62); ACC > CL > PtA (1.92); ACC > CL > V1/V2 (2.49); plPFC > CL > ACC (2.60); plPFC > CL > plPFC (2.47); plPFC > CL > PtA (3.91); and plPFC > CL > V1/V2 (2.75).

Based on the average APs/light pulse metric, the statistically significant clustered type I circuits included ACC > CL > PtA (1.09); ACC > CL > V1/V2 (0.85); plPFC > CL > PtA (1.94); and plPFC > CL > V1/V2 (1.27). APs/light pulse clustered type II circuits included ACC > CL > ACC (1.65); ACC > CL > plPFC (1.66); ACC > CL > PtA (1.46); ACC > CL > V1/V2 (1.45); plPFC > CL > ACC (0.93); plPFC > CL > PtA (1.46); and plPFC > CL > V1/V2 (2). Notably, while the ilPFC > CL > RSC circuit (Figures 4A and 5A) exhibited connectivity, it did not reach statistical significance for clustering into ACC- and plPFC-originating circuits.

Lastly, we compared the postsynaptic responses of type I versus type II neurons for each of the 35 circuits tested (Figure 5D). To account for the burst-firing properties of type II neurons, we transformed the data into binary responses (0 = no APs and 1 = at least one AP) and averaged across all light intensities. Using this metric, we discovered a total of 10 circuits in which type II neurons responded more to input activation than type I neurons: ACC > CL > ACC; ACC > CL > plPFC; ACC > CL > ilPFC, ACC > CL > PtA; ACC > CL > V1/V2; ACC > CL > OFC; plPFC > CL > V1/V2; ilPFC > CL > ilPFC; ilPFC > CL > RSC; and OFC > CL > RSC. Most circuits that preferentially drove AP firing in type II neurons over type I neurons were from ACC-originating circuits. No circuits tested preferentially activated type I neurons significantly more than type II neurons.

## DISCUSSION

Mouse fMRI revealed frontal cortical functional connectivity with claustrum and with posterior sensory and association cortices, suggesting underlying synaptic connectivity. ChR2-assisted circuit mapping experiments uncovered two types of cortico-claustro-cortical circuits: homoloquial circuits, which originate from frontal cortical regions and relay back to frontal regions, and heteroloquial circuits, which originate in frontal cortices and relay to posterior cortices. We found that the two physiologically distinct subtypes of claustrum projection neurons, which are also morphologically distinct, differentially support *trans*-claustral cortico-claustro-cortical circuits. However, frontal cortical inputs onto claustrum projection neurons also differ in strength depending on the output region of a given claustrum neuron regardless of subtype. These data indicate that the claustrum is synaptically configured to allow for flow of information from frontal cortices back to frontal cortices, as well as to posterior cortices, in a circuit- and cell-type-specific manner that reflects whole-brain-imaging functional connectivity data.

Like cortico-basal ganglia-cortical and cortico-thalamo-cortical pathways, the present data describe an extended cortical system involving significant cortical input to a subcortical nucleus—the claustrum—that returns processed information back to cortex. The claustrum appears situated to provide cortical input back not only to originating frontal cortical nuclei but to areas that are distant from frontal cortices, including posterior cortical structures. Higher-order thalamic structures, such as the lateral posterior nuclei (putative pulvinar homolog in mouse) and the mediodorsal nucleus, receive converging input from layer 5/6 prefrontal and sensory cortical projection neurons,^41–43^ which in turn propagate incoming signals back to superficial layers 2/3 of the prefrontal cortex^41,44^ with some innervation of layer 1 interneurons.^45^ This contrasts with the claustrum, which projects to layers 2/3, 5, and 6 in frontal cortices in mouse^27,46^ and predominantly layers 4/6 in sensory regions, as reported in cat.^47^ These innervation differences highlight the unique contributions to cortical processing these systems provide.

The common claustro-cortical input to both frontal and posterior cortices positions the claustrum to coordinate inter-areal cortical activity. Indeed, Narikiyo and colleagues^48^ showed that mouse claustrum activation synchronizes widespread cortical activity.^48^ Thus, the major extended cortical communications network revealed herein, together with findings that the claustrum is functionally connected with human cortical networks,^19,20^ supports the notion that the claustrum may be a central subcortical support system for cortical network function. Moreover, psilocybin, an agonist of serotonin 2A receptors, which are highly expressed in the claustrum,^49^ disrupts claustrum activity, cortical network integrity, and claustrum functional connectivity with cortical networks in human subjects.^20^

An interesting observation in the electrophysiology data was the strong ilPFC > CL > RSC synaptic connectivity strength, which occurred predominantly through type II neurons. This contrasted with the plPFC > CL > RSC results: many RSC-projecting claustrum neurons hyperpolarized in response to plPFC afferent stimulation. Further, hyperpolarizing responses were observed upon plPFC input activation of claustrum neurons projecting to the ilPFC, and vice versa. This contrast between ilPFC- and plPFC-driving circuits may suggest that the claustrum supports discrete network states. Speculatively, this is interesting considering that (1) human task-positive and default mode cortical networks are anti-correlated^33,50,51^; (2) RSC is identified as a putative node of the default mode network in mouse^52^; and (3) the plPFC and PtA are putative mouse homologs of nodes in the frontoparietal network (dorsal lateral prefrontal cortex and posterior parietal cortex, respectively).^53,54^ As such, the present data may support the idea of the claustrum acting as a relay system sculpting defined cortical networks.

Our data suggest that the claustrum differentially relays frontal cortical signals in a claustrum projection neuron subtype-dependent manner. For example, we found that inputs arising from the ACC preferentially activate type II neurons significantly more than type I neurons. Although further work is needed to define the functional significance of these pathways, we speculate that since synchronized rhythms are a major hallmark of networks,^55^ an executive cortical structure may “jump start” specific networks states through the burst-firing properties of type II claustrum neurons. This notion fits with recent data showing that claustrum neuron ensembles are not modulated by “bottom-up” sensory inputs but rather are synchronized to preferring contralateral licks in a sensory selection task.^56^ Given the transient firing nature of claustrum projection neurons^27^ and the ability for single claustrum neurons to project to multiple functionally related brain regions,^57^ the claustrum may function to switch, but not maintain, cortical network states upon cognitive demand. This is supported by the finding that significant claustrum activation is observed when task-negative networks diminish and task-positive networks emerge at the beginning of a complex cognitive task.^19^

The present findings do not rule out the existence of other cortico-claustro-cortical or sub-cortical-claustro-cortical pathways. However, considering that the majority of input to the claustrum is cortical and that frontal inputs drive APs in the claustrum, as opposed to sensory inputs,^18^ the present results highlight what is likely the bulk of information flow through claustrum. While the connections defined here support cortical network motif architectures, they also suggest that frontal cortical areas may communicate with one another through the claustrum, perhaps for dynamic control of downstream network states. Taken together with the thalamic nuclei and cortico-cortical connections that support putative default mode network connectivity in mouse,^34^ the cortical source, claustrum cell-type-, and cortical target-specific pathways defined here all likely cooperate to coordinate inter-areal cortical activity for optimal cognitive performance.

### Limitations of the study

Spatial resolution limits the anatomical accuracy of fMRI, particularly in small animals. This, coupled with the fact that fMRI and synaptic connectivity mapping approaches performed herein sample different types of data, means that the fMRI and synaptic connectivity datasets should be considered correlational. While the ChR2-assisted circuit mapping approach used is powerful, we observed a few instances of retrogradely labeled claustrum neurons arising from our ChR2-expressing anterograde virus injections, particularly from injections into the ACC and the OFC. This suggests that some excitation of recorded claustrum projections might be due to ChR2 expression in recorded neurons or due to polysynaptic excitation from neighboring ChR2-positive claustrum projection neurons. However, we failed to observe AP firing from every neuron in ACC- and OFC-originating circuits every time light was delivered (as would be expected if recording from a ChR2-positive neuron), and a previous study showed that the probability of claustrum projection neuron-to-claustrum projection neuron connection is low (~2% of total paired recordings tested).^58^ Thus, the current report of circuit-specific variable synaptic connectivity strength through the claustrum provides empirical evidence for a role of the claustrum in synaptically propagating contralateral frontal cortical information to downstream cortical targets in a manner reflective of cortical network motifs.

## STAR★METHODS

Detailed methods are provided in the online version of this paper and include the following:

### RESOURCE AVAILABILITY

#### Lead contact

Further information and requests for resources and reagents should be directed to and will be fulfilled by the lead contact, Dr. Brian N. Mathur (bmathur@som.umaryland.edu).

#### Materials availability

This study did not generate new unique reagents. Information on viruses and reagents used in this study are available in the key resources table.

#### Data and code availability

- Averaged raw data are available in tabular form (Table 1) and in Figure S2.
- This paper does not use, and therefore does not report, original code.
- Any additional information required to reanalyze the data reported in this paper is available from the lead contact upon request.

### EXPERIMENTAL MODEL AND SUBJECT DETAILS

#### Animals

5 C57BL/6 (wild type) mice of both sexes were used for neuron 3D reconstruction experiments. 40 wild-type male and female mice were used for circuit histology experiments. 175 male and female wild-type mice were used for all *ex vivo* ChR2 circuit mapping whole-cell patch clamp experiments (5 mice per circuit). A total of ~10 mice were not included in the final sample size due to contaminating virus expression in neighboring cortical areas or lack of expression. Mice used for all *ex vivo* experiments were 12–16 weeks of age and were group-housed with food and water available *ad libitum* and on a 12 h day/night light cycle beginning at 07:00 and all patch-clamp experiments were performed during the light cycle. This study was performed in accordance with the National Institutes of Health Guide for Care and Use of Laboratory Animals and the University of Maryland, School of Medicine, Animal Care and Use Committee.

### METHOD DETAILS

#### Stereotaxic procedures and viral vectors

Mice were anesthetized via inhalation of 3.5% isoflurane and placed in a mouse stereotaxic frame while anesthesia was maintained with 1% isoflurane inhalation. A stereotaxic drill was used to drill small openings in the mouse skull above brain regions prior to viral injection. 250nL of an anterograde adeno-associated virus (AAV) vector expressing a green fluorescent protein under the *hSyn* (human synapsin) promoter (AAV5-hSyn-eGFP; Addgene) was injected into ACC to fluorescently mark the anatomical boundary of the claustrum^40,59^ in order to cell fill spiny claustrum projection neurons for 3D reconstruction analysis. Relative to bregma, the coordinates used for ACC injections were anterior-posterior (AP): +1.0mm, medial-lateral (ML): ±0.3mm, dorsal-ventral (DV): −1.1mm. For all slice electrophysiology experiments, 200nL injections into the input nucleus were performed bilaterally using an AAV vector expressing ChR2 (AAV5-hSyn-ChR2-eYFP; Addgene) and simultaneously injected 150nL of a retrograde AAV expressing a td Tomato tag under the *CAG* (chicken beta-actin) promoter (rgAAV-CAG-td tomato; Addgene)^60^ into the output nucleus to fluorescently label claustrum projection neurons projecting to the target region. Exactly 4 weeks of virus incubation was given before mice were euthanized and brain slices were taken for *ev vivo* cellular recordings. Coordinates for the following brain regions were used for slice electrophysiology experiments: ACC: (see above); plPFC: (AP = +2.0mm, ML = ±0.4mm, DV = −1.2mm); ilPFC: (AP = +1.78, ML = ±0.3mm, DV = −2.2mm) OFC: (AP = +2.6mm, ML = ±1.1mm, DV = −1.8mm); aINS: (AP = +1.94mm, ML = ±2.5mm, DV = −3.5mm); PtA: (AP = −1.9mm, ML = ±1.4mm, DV = −0.4mm); V1/V2: (AP = −2.9mm, ML = ±2.05mm, DV = −0.4mm); RSC: (AP = −1.6mm, ML = ±0.3mm, DV = −0.5mm). DV coordinates were measure from top of brain surface. All injection sites were checked for accuracy before proceeding with experimentation.

#### Histology

Mice were overdosed on isoflurane gas and perfused with room temperature 0.1M phosphate-buffered solution (PBS), pH 7.3, and then with ice-cold 4% paraformaldehyde (PFA) solution in PBS, 10 days after viral injection surgery. After extraction, the brains were post-fixed in 4% PFA solution overnight. 50 μm thickness slices were obtained using the Integraslice 7550 MM vibrating microtome (Campden Instruments, Loughborough, England), and were stored at 4°C in 0.1M PBS. The slices were mounted onto 25 × 75 × 1 mm frosted microscope slides (Thermo-Scientific, Waltham, MA, United States) using 125 uL ProLong Gold antifade reagent (Invitrogen) as the mountant. The slides were imaged using a Nikon fluorescence microscope (Nikon, Minato, Tokyo, Japan) with images obtained using both 4X and 10X magnification objectives.

#### Resting state functional connectivity fMRI analysis

Mouse resting state fMRI data was obtained from a publicly available dataset (https://public.data.donders.ru.nl/dcmn/DSC_4180000.18_502_v1, https://public.data.donders.ru.nl/dcmn/DSC_4180000.18_502_v1/LICENSE.txt). The specific data used consisted of scans from an experiment testing the effects of a model of psychosocial stress on male, wild-type, C57BL/6 mice aged 3 months.^61^ We therefore used only pre-intervention baseline scans from all 51 animals, both control and experimental, available online.

Full details on data acquisition are described in a previous fMRI study.^61^ Anesthesia was induced with 3.5% isoflurane, mice were ventilated at 80 breaths per minute, and anesthesia was maintained with a combination of pancuronium bromide, medetomidine, and a gradual reduction to 0.5% isoflurane. Scans were acquired with a Bruker 94/30 Biospec spectrometer (Bruker BioSpin MRI, Ettlingen, Germany) operating at 9.4 T. Resting state fMRI scans consisted of 6 min of blood oxygenation level-dependent (BOLD) gradient-echo echo planar images acquired using repetition time TR = 1000 ms, echo time TE = 9.2 ms, flip angle FA = 90°, matrix size MS = 90 × 70, field of view FOV = 20 × 17.5 mm^2^, slice number NS = 12, slice thickness ST = 0.5 mm, slice gap SG = 0.2 mm, and bandwidth BW = 250,000 Hz.

Full details on preprocessing are described in a previous study.^62^ When downloaded, the analyzed images were already preprocessed according to the protocol previously described.^62^ In brief, anatomical images were registered to the Allen Institute for Brain Science (AIBS) mouse template (https://atlas.brain-map.org/). Functional images were despiked (3dDespike), motion-corrected (3dvolreg), corrected for B1 field, denoised, brain-masked, registered linearly to corresponding anatomical images, and bandpass filtered (3dBandpass, 0.01–0.25 Hz).

The five unilateral frontal cortical seed regions of interest (ROIs) and the contralateral claustrum were drawn using FSLeyes.^63,64^ For each ROI, the AIBS template used for preprocessing was loaded in the software, an empty 3D mask with the same dimensions was generated and overlaid on the template, and the voxels of the ROI were hand-selected using the anatomical knowledge of the authors,^65^ and anatomical landmarks visible in the AIBS template. The seed and claustrum ROIs as drawn can be seen in Figure 1A.

Analyses were performed in SPM12. To assess whole brain functional connectivity of the seed regions, the SPM toolbox MarsBar was used to extract each animal’s mean BOLD signal timeseries from each seed ROI, and individual General Linear Models were produced in SPM for each animal/ROI consisting of the mean ROI timeseries and 6 motion parameters as regressors.

To determine significant functional connectivity, one sample t-tests were performed on resting state second-level contrast maps masked with the AIBS template binary mask. To correct for multiple comparisons, we imposed an FWE-corrected voxel-wise significance threshold of p < 0.05. Data are publicly available, and code and ROI files are available upon request.

#### Three-dimensional reconstructions of claustrum spiny projection neurons

Type I and type II claustrum projection neurons were recorded under whole-cell current clamp conditions and classified based on their burst firing properties, or lack thereof,^27^ following a brief 2ms depolarizing current injection step. Respective neurons were recorded with a potassium-based solution (290–295 mOsm; pH) with 5% concentration biocytin to allow for proper cell fill into the soma and distal dendrites. Immediately following electrophysiological recording, slices were fixed in 4% paraformaldehyde overnight at 4°C. The next day, slices were washed in 0.1 M phosphate buffered saline (PBS) 3 × 20 min and blocked with 1% BSA in PBS +0.3% Triton X-100 (PBS-T) for 2 h at room temperature. Slices were incubated with Alexa Fluor 594-streptavidin (Invitrogen, #S32356) (1:1000, 1% BSA in 0.3% PBS-T) overnight at 4°C. The next day, slices were washed in PBS 3 × 20 min, mounted on slides, and coverslipped in ProLong Diamond Antifade (Invitrogen, #P36965).

Confocal images were acquired with a Nikon A1 microscope equipped with 488 nm and 561 nm lasers. For neuronal reconstructions, slices were first imaged for both GFP and Alexa Fluor 594 expression to verify the position of each neuron within the claustrum. Neurons were then imaged using a 40x (0.95 NA) objective with a lateral resolution of 0.310 μm per pixel and a 0.727 μm z-step. For dendritic spine reconstruction and quantification, two dendrites were imaged per cell using a 100x (1.46 NA) oil-immersion objective with a lateral resolution of 0.05 μm per pixel and a 0.10 μm z-step. Dendrites were randomly chosen, taking care to avoid broken or proximal (<50 μm from cell body) sections, and a roughly 50 μm section was imaged for analysis. Prior to spine analysis, raw images were denoised using the Nikon Elements Denoise.ai algorithm.

Semi-automated neuronal reconstruction was performed using Neurolucida 360 (version 2020.1.1) with directional kernels algorithm. Dendritic spine analysis was conducted using a semi-automated analysis method using Bitplane Imaris (version 9.5.1). The Filaments module was used to first reconstruct sections of dendrite then detect spines. Spine detection was edited for accuracy when necessary by an experimenter blind to cell type.

#### *Ex vivo* brain slice preparation for slice electrophysiology

Male and female wildtype C57BL/6 Mice between the ages of 5–8 weeks were surgically injected with both viruses bilaterally and following four weeks of virus incubation were euthanized for *ex vivo* recordings. Following anesthetization, mice were decapitated, and the brains were extracted. 250 μm coronal sections were sliced using a Leica VT1200 vibrating microtome in a high-sucrose artificial cerebrospinal fluid (aCSF) solution. The high-sucrose cutting aCSF solution was kept ice-cold, continuously bubbled with carbogen (95% O_2_, 5% CO_2_), and was comprised of 194 mM sucrose, 30 mM NaCL, 4.5 mM KCL, 1 mM MgCl_2_, 26 mM NaHCO_3_, 1.2 mM NaH_2_PO_4_, and 10 mM D-glucose. Sections were incubated after slicing for 30 min at 33°C in carbogen-bubbled aCSF (315–320 mOsm) that contained 124 mM NaCl, 4.5 mM KCl, 2 mM CaCl_2_, 1 mM MgCl_2_, 26 mM NaHCO_3_, 1.2 mM NaH_2_PO_4_, and 10 mM D-glucose. Brain slices were incubated at room temperature until whole-cell patch-clamp recordings, and patch recordings were performed in the same aCSF formulation used for incubation. We monitored cell health and recording quality before and after optogenetic stimulation by examining resting membrane potential, access resistance and membrane resistance. If resting membrane potential changed by more than 10 mV we discarded the cell. If the resting membrane potential began more depolarized than the average range for claustrum projection neurons the cell was discarded.^66^ If access resistance and membrane resistance changed by more than 15% we discarded the cell.

#### Whole-cell current clamp electrophysiological recordings

Whole-cell recordings were performed at 29–31°C using borosilicate glass recording pipettes of 3–7Ω MU resistance. For recordings performed in a current clamp configuration, recording pipettes were filled with a potassium-based solution (290–295 mOsm; pH 7.3) composed of 126 mM potassium gluconate, 4 mM KCl, 10 mM HEPES, 4 mM ATP-Mg, 0.3 mM GTP-Na and 10 mM phosphocreatine. Clampex software (Version 10.4; Molecular Devices) was used for all electrophysiological recordings. Recordings were filtered at 2 kHz and digitized at 10 kHz using MultiClamp 700B software (Molecular Devices). Claustrum projection neuron type was determined via a 5 ms depolarization step while recording in current-clamp mode to determine burst firing properties (Type I: no burst fire; Type II: burst fire). Following this protocol, membrane capacitance values were also recorded to confirm the characterization of neuron type (Type I: ~75–130 pF; Type II: ~130–200 pF).^27^ For all slice electrophysiology experiments, three 5 ms 470nm blue light pulses with 150 ms intervals were given to evoke presynaptic transmitter release while recording from fluorescently labeled claustrum projections.^67^

### QUANTIFICATION AND STATISTICAL ANALYSIS

#### Circuit mapping data analysis and statistics

Three 5ms light pulses were delivered to optically stimulate ChR2-expressing glutamatergic afferents arising from a given cortical region to drive postsynaptic responses. To quantify the degree of the postsynaptic response in each circuit, we used: 1) average action potentials (APs) per light stimulation intensity and 2) area under the curve (AUC) for each postsynaptic recording trace. For AUC, a higher positive AUC value represents a postsynaptic depolarization, reflecting an excitatory postsynaptic potential (EPSP) or an AP. Conversely, a negative AUC value reflects a postsynaptic hyperpolarization. Notably, using the same potassium-based internal solution used throughout, we found that the reversal potential for inhibitory synaptic currents at both type I and II neuron synapses was −75mV (Figure S3). Electrophysiology data were analyzed using Clampex software (version 11.0.3). Area under the curve values were converted from voltage values for each trace in Clampex data table files into excel files. Values for each circuit, cell, and light intensity were analyzed using MATLAB (version 2019a) using trapezoidal integration for area under the curve to access activation/inactivation of each cell following presynaptic stimulation. Representative heatmaps were averaged using Microsoft excel and plotted in MATLAB. Multiple comparison statistical analyses were performed using GraphPad Prism software (Prism 8). A nonparametric test with multiple comparisons was used to compare average AUC and actional potential values for all 35 circuits (Figure S5). Subgraph extraction analyses was performed by determining the p value of a subgraph through the probability to obtain the subgraph under the null hypothesis that none of the circuits and edges are significant (approximated by permutation test). Cohen’s d values for all the data points were calculated using MATLAB. All statistical details of experiments, sample sizes, and precision measures can be found in the figure legends.

## Supplementary Material

1

## Figures and Tables

**Table 1.** *Ex vivo* cortico-claustral-cortical circuit mapping average area under the curve and number of action potentials per light pulse values

Outputs								

Inputs	Type I							
	
		ACC	plPFC	ilPFC	OFC	RSC	V1/V2	PtA
	
	ACC	980.57 ± 234.03	1,726.36 ± 500.26	874.5 ± 158.12	365.18 ± 97.84	543.90 ± 100.59	1,187.82 ± 339.33	2,612.2 ± 499.74
	
	pIPFC	1,110.11 ± 329.41	829.25 ± 283.61	−126.12 ± 75.50	910.73 ± 253.03	−5.03 ± 37.58	2,063.42 ± 642.18	2,857.86 ± 1,006.19
	
	ilPFC	90.03 ± 10.76	−87.94 ± 34.25	418.33 ± 82.48	921.15 ± 214.65	948.56 ± 203.32	585.93 ± 95.61	425.83 ± 69.29
	
	OFC	558.56 ± 120.27	391.55 ± 72.17	437.82 ± 79.15	334.97 ± 56.41	260.83 ± 40.83	514.24 ± 135.05	318.96 ± 51.90
	
	aINS	133.14 ± 24.28	126.87 ± 25.88	361.20 ± 66.63	170.44 ± 20.30	259.51 ± 46.23	97.69 ± 21.15	261.55 ± 54.64
	
	Type II							
	
		ACC	plPFC	ilPFC	OFC	RSC	V1/V2	PtA
	
	ACC	1,888.64 ± 621.51	2,283.24 ± 718.96	1,630.08 ± 437.59	1,092.71 ± 369.70	1,018.83 ± 228.81	1,387.44 ± 427.60	1,846.68 ± 611.11
	
	pIPFC	1,448.17 ± 422.10	1,397.84 ± 400.05	257.79 ± 33.61	711.19 ± 171.64	904.83 ± 172.07	2,963.15 ± 972.68	2,698.31 ± 997.09
	
	ilPFC	393.51 ± 55.72	367.92 ± 66.75	1,521.39 ± 379.03	646.72 ± 141.09	3,024.88 ± 900.08	974.24 ± 205.79	1,208.80 ± 261.53
	
	OFC	645.47 ± 158.26	496.05 ± 103.38	590.06 ± 118.20	576.29 ± 143.41	1,006.56 ± 288.62	634.90 ± 218.70	495.16 ± 158.10
	
	aINS	137.10 ± 14.48	114.51 ± 23.34	196.16 ± 21.01	222.86 ± 21.19	257.16 ± 44.34	150.73 ± 18.56	273.46 ± 93.64
	
Outputs								

Inputs	Type I							
	
		ACC	plPFC	ilPFC	OFC	RSC	V1/V2	PtA
	
	ACC	0.20 ± 0.09	0.11 ± 0.06	0.03 ± 0.01	0.00 ± 0.00	0.11 ± 0.05	0.14 ± 0.08	0.19 ± 0.10
	
	pIPFC	0.11 ± 0.07	0.10 ± 0.05	0.00 ± 0.00	0.04 ± 0.02	0.00 ± 0.00	0.36 ± 0.12	0.40 ± 0.15
	
	ilPFC	0.00 ± 0.00	0.00 ± 0.00	0.06 ± 0.03	0.02 ± 0.01	0.10 ± 0.04	0.01 ± 0.01	0.01 ± 0.01
	
	OFC	0.05 ± 0.03	0.00 ± 0.00	0.00 ± 0.00	0.00 ± 0.00	0.01 ± 0.00	0.00 ± 0.00	0.00 ± 0.00
	
	aINS	0.00 ± 0.00	0.00 ± 0.00	0.00 ± 0.00	0.00 ± 0.00	0.00 ± 0.00	0.00 ± 0.00	0.00 ± 0.00
	
	Type II							
	
		ACC	plPFC	ilPFC	OFC	RSC	V1/V2	PtA
	
	ACC	0.55 ± 0.21	0.55 ± 0.23	0.52 ± 0.18	0.14 ± 0.09	0.13 ± 0.07	0.46 ± 0.18	0.48 ± 0.22
	
	pIPFC	0.26 ± 0.12	0.30 ± 0.14	0.00 ± 0.00	0.05 ± 0.03	0.02 ± 0.01	1.04 ± 0.36	0.70 ± 0.26
	
	ilPFC	0.00 ± 0.00	0.01 ± 0.00	0.32 ± 0.11	0.09 ± 0.03	0.78 ± 0.38	0.07 ± 0.03	0.09 ± 0.04
	
	OFC	0.01 ± 0.01	0.00 ± 0.00	0.00 ± 0.00	0.02 ± 0.01	0.11 ± 0.06	0.01 ± 0.01	0.02 ± 0.01
	
	aINS	0.00 ± 0.00	0.00 ± 0.00	0.00 ± 0.00	0.00 ± 0.00	0.00 ± 0.00	0.00 ± 0.00	0.00 ± 0.00

Top: average area under the curve values across all light intensities tested (0, 0.6, 1.2, 1.8, 2.4, and 3 mW) for all type I and II claustrum neurons tested (n = 1,050 cells). Bottom: average number of action potentials per light pulse values across all light intensities. Error shown is standard error of the mean. Average area under the curve units = mV × ms.

**KEY RESOURCES TABLE:** 

REAGENT or RESOURCE	SOURCE	IDENTIFIER

Antibodies		

Alexa Fluor 594-streptavidin	Invitrogen	#S32356; RRID: SCR_008410

Bacterial and virus strains		

AAV5-hSyn-eGFP	Brian Roth/Addgene	#50465-AAV5 RRID: Addgene_50465
pAAV5-hSyn-hChR2(H134R)-EYFP	Karl Deisseroth/Addgene	#26973-AAV5 RRID: Addgene_26973
rgAAV-CAG-td tomato	Edward Boyden/Addgene	#59462-AAVrg RRID: Addgene_59462

Experimental models: Organisms/strains		

Mouse, C57BL/6J	Jackson Laboratory	#000664

Software and algorithms		

Data analysis: Python 3.9	Python Software Foundation	https://www.python.org
Data analysis: Prism 8.01	GraphPad	https://www.graphpad.com
Clampex Suite 11.0.3	Molecular Devices	https://www.moleculardevices.com
MATLAB 2019a	Mathworks	https://www.mathworks.com
Neurolucida 360 2020.1.1	MBF Bioscience	https://mbfbioscience.com
Bitplane Imaris 9.5.1	Oxford Instruments	https://imaris.oxinst.com/
fMRI ROI analysis: FSLeyes	University of Oxford	https://fsl.fmrib.ox.ac.uk/fsl/fslwiki/FSLeyes

Other		

Mouse rsfMRI dataset used in paper	Grandjean et al., 2016^61^	https://public.data.donders.ru.nl/dcmn/DSC_4180000.18_502_v1

## Highlights

- Frontal cortices are functionally connected with claustrum and posterior cortices
- Frontal cortices are synaptically connected to posterior cortices through claustrum
- Two claustrum projection neuron subtypes support *trans*-claustral circuits
- *Trans*-claustral circuits resemble a frontoposterior cortical network motif
