# Supplementary material for: The mouse claustrum synaptically connects cortical network motifs
Source: Cell Rep. Author manuscript; Available in PMC 2023 Jan 13. (PMC9838879; doi:10.1016/j.celrep.2022.111860)
Supplement: 1 [file NIHMS1860007-supplement-1.pdf]

Cell Reports, Volume *41*

Supplemental information

The mouse claustrum synaptically

connects cortical network motifs

Houman Qadir, Brent W. Stewart, Jonathan W. VanRyzin, Qiong Wu, Shuo Chen, David A. Seminowicz, and Brian N. Mathur

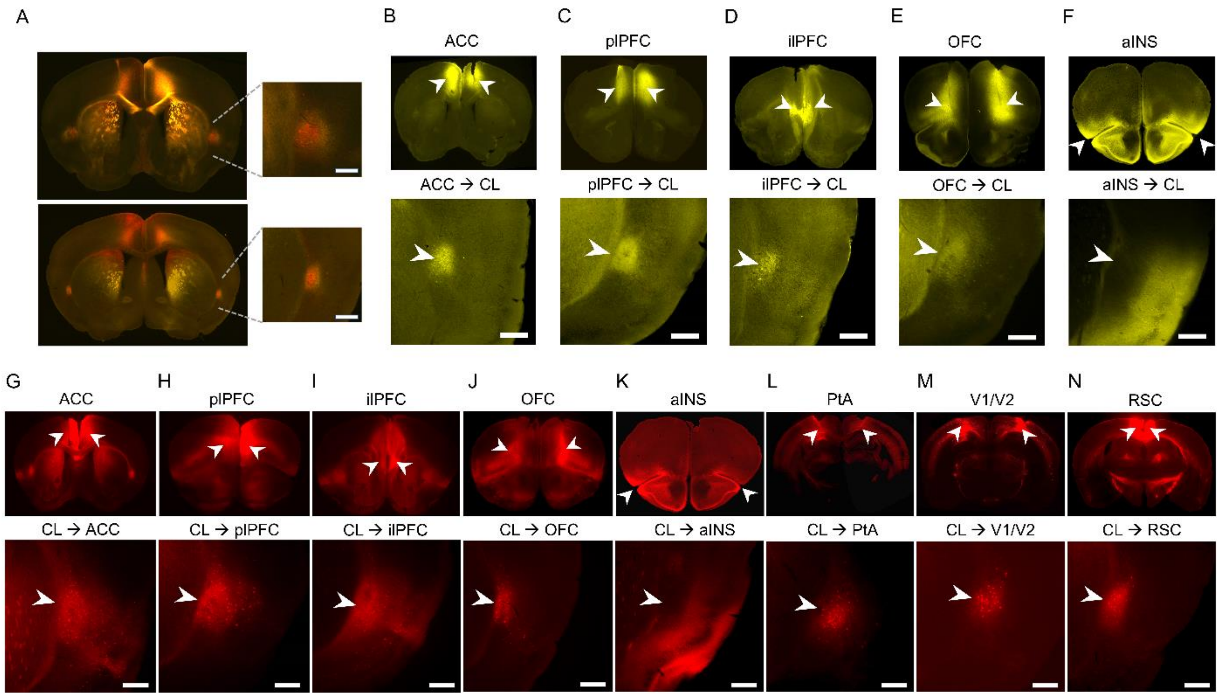

Figure S1. Structural connectivity of putative frontal cortico-claustral-cortical circuits. Related to Figure 3.

A) Representative merged image of anterograde ChR2-eYFP virus and retrograde td-Tomato expression in the claustrum (Top: anterior claustrum section; Bottom: posterior claustrum section). Representative circuit shown: plPFC > CL > PtA. B-F) Top panels: representative photomicrographs of anterograde eYFP virus injection sites in the B) ACC, C) plPFC, D) ilPFC, E) OFC, and F) aINS. Bottom panels: representative photomicrographs of anterograde terminal expression in the claustrum from respective frontal regions. Structural inputs were strongest from the ACC and plPFC with moderate inputs from the OFC. Very weak inputs were observed from the aINS based on anterograde viral expression in the claustrum. G-N) Top panels: representative photomicrographs of retrograde td-Tomato virus injection sites in the ACC (G), plPFC

(H), ilPFC(I), OFC (J), aINS (K), PtA (L), visual cortex (V1/V2) (M), and RSC (N). Bottom panels: representative photomicrographs of retrograde td-Tomato expression in the claustrum. N=3 per cases per circuit. Horizontal scale bars: A-N) 500µm.

A

Type I

	3.0 mW	2.4 mW	1.8 mW	1.2 mW	0.6 mW	0 mW	Mean of average AUC per light intensity
ACC CL ACC	1708.82	1466.01	1158.43	778.03	606.94	165.17	980.57 ± 234.03
ACC CL plPFC	3180.24	2896.37	2091.43	1471.25	571.49	147.38	1726.36 ± 500.26
ACC CL ilPFC	1176.31	1208.02	1105.60	886.07	668.71	202.32	874.50 ± 158.12
ACC CL OFC	750.65	555.19	309.09	264.34	184.29	127.55	365.18 ± 97.84
ACC CL RSC	821.55	763.19	637.61	505.88	355.55	179.64	543.90 ± 100.59
ACC CL V1V2	2208.17	2012.44	1430.23	813.89	513.63	148.55	1187.82 ± 339.33
ACC CL PtA	2612.24	3409.71	1636.80	1223.70	536.80	214.64	2612.20 ± 499.74
plPFC CL plPFC	1967.84	1300.69	813.08	582.90	236.41	134.55	829.25 ± 283.61
plPFC CL ACC	2310.61	1647.40	1306.61	847.98	374.35	173.71	1110.11 ± 329.41
plPFC CL ilPFC	-402.82	-261.85	-147.30	-65.96	27.10	94.11	-126.12 ± 75.50
plPFC CL OFC	1674.79	1470.59	1164.13	648.51	366.80	139.54	910.73 ± 253.03
plPFC CL RSC	110.81	-123.77	-64.49	-46.19	0.52	92.93	-5.03 ± 37.58
plPFC CL V1V2	4147.54	3409.71	2490.53	1560.95	662.12	109.67	2063.42 ± 642.18
plPFC CL PtA	6509.99	4809.25	3101.12	1977.93	545.24	203.60	2857.86 ± 1006.19
ilPFC CL ilPFC	579.94	625.80	518.92	418.59	268.25	98.51	418.33 ± 82.48
ilPFC CL ACC	136.10	101.39	79.46	67.91	65.07	90.26	90.03 ± 10.76
ilPFC CL plPFC	-187.47	-166.87	-111.74	-73.99	-10.64	23.06	-87.94 ± 34.25
ilPFC CL OFC	1403.12	1451.54	1186.92	859.13	478.46	147.71	921.15 ± 214.65
ilPFC CL RSC	1488.57	1307.60	1283.53	829.09	581.19	201.39	948.56 ± 203.32
ilPFC CL V1V2	809.74	764.93	698.62	590.94	473.86	177.48	585.93 ± 95.61
ilPFC CL PtA	643.88	530.37	502.47	392.67	318.99	166.62	425.83 ± 69.29
OFC CL OFC	473.62	488.26	371.96	302.58	243.07	130.35	334.97 ± 56.41
OFC CL ACC	917.08	783.48	703.72	494.94	291.62	160.53	558.56 ± 120.27
OFC CL plPFC	622.49	561.55	409.43	342.95	243.99	168.89	391.55 ± 72.17
OFC CL ilPFC	639.34	621.59	513.13	412.06	299.95	140.83	437.82 ± 79.15
OFC CL RSC	358.25	372.29	300.46	215.22	203.35	115.39	260.83 ± 40.83
OFC CL V1V2	984.16	805.28	538.87	384.48	253.78	118.87	514.24 ± 135.05
OFC CL PtA	456.82	432.57	378.37	295.69	216.70	133.60	318.96 ± 51.90
aINS CL ACC	219.69	170.38	126.11	145.19	81.47	56.03	133.14 ± 24.28
aINS CL plPFC	233.32	167.29	123.91	88.61	80.25	67.84	126.87 ± 25.88
aINS CL ilPFC	555.46	485.07	441.92	327.93	231.50	125.31	361.20 ± 66.63
aINS CL OFC	241.38	202.87	192.21	145.11	130.01	111.07	170.44 ± 20.30
aINS CL RSC	420.31	336.05	275.27	243.35	184.79	97.28	259.51 ± 46.23
aINS CL V1V2	195.99	93.01	106.45	61.78	73.39	55.53	97.69 ± 21.15
aINS CL PtA	483.67	306.81	290.86	223.21	164.73	98.84	261.55 ± 54.64

Type I

	3.0 mW	2.4 mW	1.8 mW	1.2 mW	0.6 mW	0 mW	Mean of average APs per light intensity
ACC CL ACC	0.53	0.38	0.22	0.09	0.00	0.00	0.20 ± 0.09
ACC CL plPFC	0.35	0.22	0.07	0.00	0.00	0.00	0.11 ± 0.06
ACC CL ilPFC	0.07	0.07	0.02	0.00	0.00	0.00	0.03 ± 0.01
ACC CL OFC	0.02	0.00	0.00	0.00	0.00	0.00	0.00 ± 0.00
ACC CL RSC	0.27	0.24	0.13	0.04	0.00	0.00	0.11 ± 0.05
ACC CL V1V2	0.49	0.24	0.11	0.02	0.00	0.00	0.14 ± 0.08
ACC CL PtA	0.60	0.42	0.11	0.02	0.00	0.00	0.19 ± 0.10
plPFC CL plPFC	0.31	0.20	0.04	0.02	0.00	0.00	0.10 ± 0.05
plPFC CL ACC	0.40	0.24	0.04	0.00	0.00	0.00	0.11 ± 0.07
plPFC CL ilPFC	0.00	0.00	0.00	0.00	0.00	0.00	0.00 ± 0.00
plPFC CL OFC	0.16	0.07	0.04	0.00	0.00	0.00	0.04 ± 0.02
plPFC CL RSC	0.00	0.00	0.00	0.00	0.00	0.00	0.00 ± 0.00
plPFC CL V1V2	0.67	0.67	0.53	0.22	0.02	0.07	0.36 ± 0.12
plPFC CL PtA	0.91	0.67	0.58	0.24	0.00	0.00	0.40 ± 0.15
ilPFC CL ilPFC	0.13	0.13	0.04	0.02	0.00	0.00	0.06 ± 0.03
ilPFC CL ACC	0.00	0.00	0.00	0.00	0.00	0.00	0.00 ± 0.00
ilPFC CL plPFC	0.00	0.00	0.00	0.00	0.00	0.00	0.00 ± 0.00
ilPFC CL OFC	0.09	0.02	0.00	0.00	0.00	0.00	0.02 ± 0.01
ilPFC CL RSC	0.22	0.22	0.13	0.02	0.00	0.00	0.10 ± 0.04
ilPFC CL V1V2	0.04	0.02	0.00	0.00	0.00	0.00	0.01 ± 0.01
ilPFC CL PtA	0.04	0.02	0.00	0.00	0.00	0.00	0.01 ± 0.01
OFC CL OFC	0.00	0.00	0.00	0.00	0.00	0.00	0.00 ± 0.00
OFC CL ACC	0.13	0.13	0.02	0.00	0.00	0.00	0.05 ± 0.03
OFC CL plPFC	0.00	0.00	0.00	0.00	0.00	0.00	0.00 ± 0.00
OFC CL ilPFC	0.00	0.00	0.00	0.00	0.00	0.00	0.00 ± 0.00
OFC CL RSC	0.02	0.02	0.00	0.00	0.00	0.00	0.01 ± 0.00
OFC CL V1V2	0.02	0.00	0.00	0.00	0.00	0.00	0.00 ± 0.00
OFC CL PtA	0.00	0.00	0.00	0.00	0.00	0.00	0.00 ± 0.00
aINS CL ACC	0.00	0.00	0.00	0.00	0.00	0.00	0.00 ± 0.00
aINS CL plPFC	0.00	0.00	0.00	0.00	0.00	0.00	0.00 ± 0.00
aINS CL ilPFC	0.00	0.00	0.00	0.00	0.00	0.00	0.00 ± 0.00
aINS CL OFC	0.00	0.00	0.00	0.00	0.00	0.00	0.00 ± 0.00
aINS CL RSC	0.00	0.00	0.00	0.00	0.00	0.00	0.00 ± 0.00
aINS CL V1V2	0.00	0.00	0.00	0.00	0.00	0.00	0.00 ± 0.00
aINS CL PtA	0.00	0.00	0.00	0.00	0.00	0.00	0.00 ± 0.00

B

Type II

	3.0 mW	2.4 mW	1.8 mW	1.2 mW	0.6 mW	0 mW	Mean of average AUC per light intensity
ACC CL ACC	4125.05	3205.33	1896.77	1293.58	629.14	181.98	1888.64 ± 621.51
ACC CL plPFC	4532.92	3959.15	2732.60	1610.04	688.32	176.43	2283.24 ± 718.96
ACC CL ilPFC	2601.49	2662.92	2334.39	1393.09	647.09	141.52	1630.08 ± 437.59
ACC CL OFC	2415.66	1868.47	1189.15	640.32	310.29	132.37	1092.71 ± 369.70
ACC CL RSC	1595.11	1544.67	1287.42	802.45	738.96	144.37	1018.83 ± 228.81
ACC CL V1V2	2859.27	2289.98	1569.94	909.74	549.54	146.17	1387.44 ± 427.60
ACC CL PtA	4187.80	2702.14	2188.80	1336.48	450.47	214.38	1846.68 ± 611.11
plPFC CL plPFC	2786.04	2172.76	1543.62	1159.87	522.56	202.17	1397.84 ± 400.05
plPFC CL ACC	2942.69	2182.47	1656.86	1212.29	504.75	189.98	1448.17 ± 422.10
plPFC CL ilPFC	366.17	302.51	269.66	262.29	226.09	120.04	257.79 ± 33.61
plPFC CL OFC	1303.56	1029.27	818.47	559.17	384.09	172.55	711.19 ± 171.64
plPFC CL RSC	1259.70	1161.57	1162.93	1018.60	671.24	154.95	904.83 ± 172.07
plPFC CL V1V2	6074.16	5431.53	3089.25	2027.24	994.06	162.65	2963.15 ± 972.68
plPFC CL PtA	6299.07	4808.10	2894.10	1385.08	622.66	180.84	2698.31 ± 997.09
ilPFC CL ilPFC	2634.10	2348.59	1743.89	1386.74	870.79	144.21	1521.39 ± 379.03
ilPFC CL ACC	524.61	492.83	455.27	410.50	323.15	154.72	393.51 ± 55.72
ilPFC CL plPFC	545.09	503.51	468.55	332.85	200.57	156.94	367.92 ± 66.75
ilPFC CL OFC	1023.20	988.03	783.13	542.27	390.95	152.73	646.72 ± 141.09
ilPFC CL RSC	5180.32	5059.25	4467.33	2460.81	863.27	118.28	3024.88 ± 900.08
ilPFC CL V1V2	1406.02	1411.32	1282.32	970.63	621.79	153.34	974.24 ± 205.79
ilPFC CL PtA	1740.57	1795.54	1539.50	1243.25	793.83	140.11	1208.80 ± 261.53
OFC CL OFC	1047.38	905.05	614.19	466.49	277.74	146.89	576.29 ± 143.41
OFC CL ACC	1133.68	1035.95	693.98	518.11	350.87	140.25	645.47 ± 158.26
OFC CL plPFC	816.10	733.07	546.86	402.19	335.32	142.79	496.05 ± 103.38
OFC CL ilPFC	920.63	843.85	690.19	537.41	402.74	145.57	590.06 ± 118.20
OFC CL RSC	1838.75	1673.24	1267.14	816.42	337.04	106.75	1006.56 ± 288.62
OFC CL V1V2	1367.79	1221.27	562.18	351.03	202.46	104.69	634.90 ± 218.70
OFC CL PtA	1090.98	843.05	424.16	284.81	215.07	112.90	495.16 ± 158.10
aINS CL ACC	194.46	150.82	132.02	124.99	134.53	85.78	137.10 ± 14.48
aINS CL plPFC	195.56	175.57	98.06	90.99	64.94	61.96	114.51 ± 23.34
aINS CL ilPFC	261.81	233.86	211.43	186.07	167.23	116.57	196.16 ± 21.01
aINS CL OFC	271.19	263.48	242.19	223.76	208.01	128.55	222.86 ± 21.19
aINS CL RSC	412.05	342.93	286.13	193.17	182.20	126.48	257.16 ± 44.34
aINS CL V1V2	206.91	190.19	169.51	129.37	119.65	88.75	150.73 ± 18.56
aINS CL PtA	684.52	385.29	235.55	134.15	122.74	78.48	273.46 ± 93.64

Type II

	3.0 mW	2.4 mW	1.8 mW	1.2 mW	0.6 mW	0 mW	Mean of average APs per light intensity
ACC CL ACC	1.13	1.09	0.73	0.33	0.04	0.00	0.55 ± 0.21
ACC CL plPFC	1.40	0.98	0.60	0.31	0.02	0.00	0.55 ± 0.23
ACC CL ilPFC	1.00	0.95	0.71	0.44	0.00	0.00	0.52 ± 0.18
ACC CL OFC	0.53	0.31	0.02	0.00	0.00	0.00	0.14 ± 0.09
ACC CL RSC	0.42	0.29	0.09	0.00	0.00	0.00	0.13 ± 0.07
ACC CL V1V2	0.96	0.91	0.69	0.20	0.00	0.00	0.46 ± 0.18
ACC CL PtA	1.20	1.07	0.47	0.18	0.00	0.00	0.48 ± 0.22
plPFC CL plPFC	0.78	0.60	0.35	0.07	0.00	0.00	0.30 ± 0.14
plPFC CL ACC	0.71	0.53	0.27	0.04	0.00	0.00	0.26 ± 0.12
plPFC CL ilPFC	0.00	0.00	0.00	0.00	0.00	0.00	0.00 ± 0.00
plPFC CL OFC	0.13	0.16	0.00	0.00	0.00	0.00	0.05 ± 0.03
plPFC CL RSC	0.07	0.02	0.02	0.00	0.00	0.00	0.02 ± 0.01
plPFC CL V1V2	2.13	1.91	1.27	0.82	0.13	0.00	1.04 ± 0.36
plPFC CL PtA	1.62	1.18	0.87	0.42	0.13	0.00	0.70 ± 0.26
ilPFC CL ilPFC	0.58	0.58	0.51	0.24	0.02	0.00	0.32 ± 0.11
ilPFC CL ACC	0.00	0.00	0.00	0.00	0.00	0.00	0.00 ± 0.00
ilPFC CL plPFC	0.02	0.02	0.00	0.00	0.00	0.00	0.01 ± 0.00
ilPFC CL OFC	0.16	0.16	0.16	0.07	0.00	0.00	0.09 ± 0.03
ilPFC CL RSC	1.95	1.82	0.87	0.04	0.00	0.00	0.78 ± 0.38
ilPFC CL V1V2	0.18	0.15	0.09	0.02	0.00	0.00	0.07 ± 0.03
ilPFC CL PtA	0.18	0.20	0.13	0.02	0.00	0.00	0.09 ± 0.04
OFC CL OFC	0.07	0.07	0.00	0.00	0.00	0.00	0.02 ± 0.01
OFC CL ACC	0.04	0.02	0.00	0.00	0.00	0.00	0.01 ± 0.01
OFC CL plPFC	0.00	0.00	0.00	0.00	0.00	0.00	0.00 ± 0.00
OFC CL ilPFC	0.00	0.00	0.00	0.00	0.00	0.00	0.00 ± 0.00
OFC CL RSC	0.33	0.22	0.09	0.00	0.00	0.00	0.11 ± 0.06
OFC CL V1V2	0.07	0.00	0.00	0.00	0.00	0.00	0.01 ± 0.01
OFC CL PtA	0.07	0.07	0.00	0.00	0.00	0.00	0.02 ± 0.01
aINS CL ACC	0.00	0.00	0.00	0.00	0.00	0.00	0.00 ± 0.00
aINS CL plPFC	0.00	0.00	0.00	0.00	0.00	0.00	0.00 ± 0.00
aINS CL ilPFC	0.00	0.00	0.00	0.00	0.00	0.00	0.00 ± 0.00
aINS CL OFC	0.00	0.00	0.00	0.00	0.00	0.00	0.00 ± 0.00
aINS CL RSC	0.00	0.00	0.00	0.00	0.00	0.00	0.00 ± 0.00
aINS CL V1V2	0.00	0.00	0.00	0.00	0.00	0.00	0.00 ± 0.00
aINS CL PtA	0.00	0.00	0.00	0.00	0.00	0.00	0.00 ± 0.00

Figure S2. *Ex vivo* circuit-mapping average area under the curve and number of action potentials per light pulse raw data. Related to Figures 4 and 5. A) Average area under the curve (AUC) values (left) and action potentials per light pulse values (right) for each light intensity tested (0-3mW) of all type I claustrum neurons (n=525 cells). B) Average AUC values (left) and actional potentials per light pulse values (right)

for each light intensity tested (0-3mW) of all type II claustrum neurons (n=525 cells). n=1,050 cells total. Error shown is standard error of the mean. Units: ** = mV x msec.

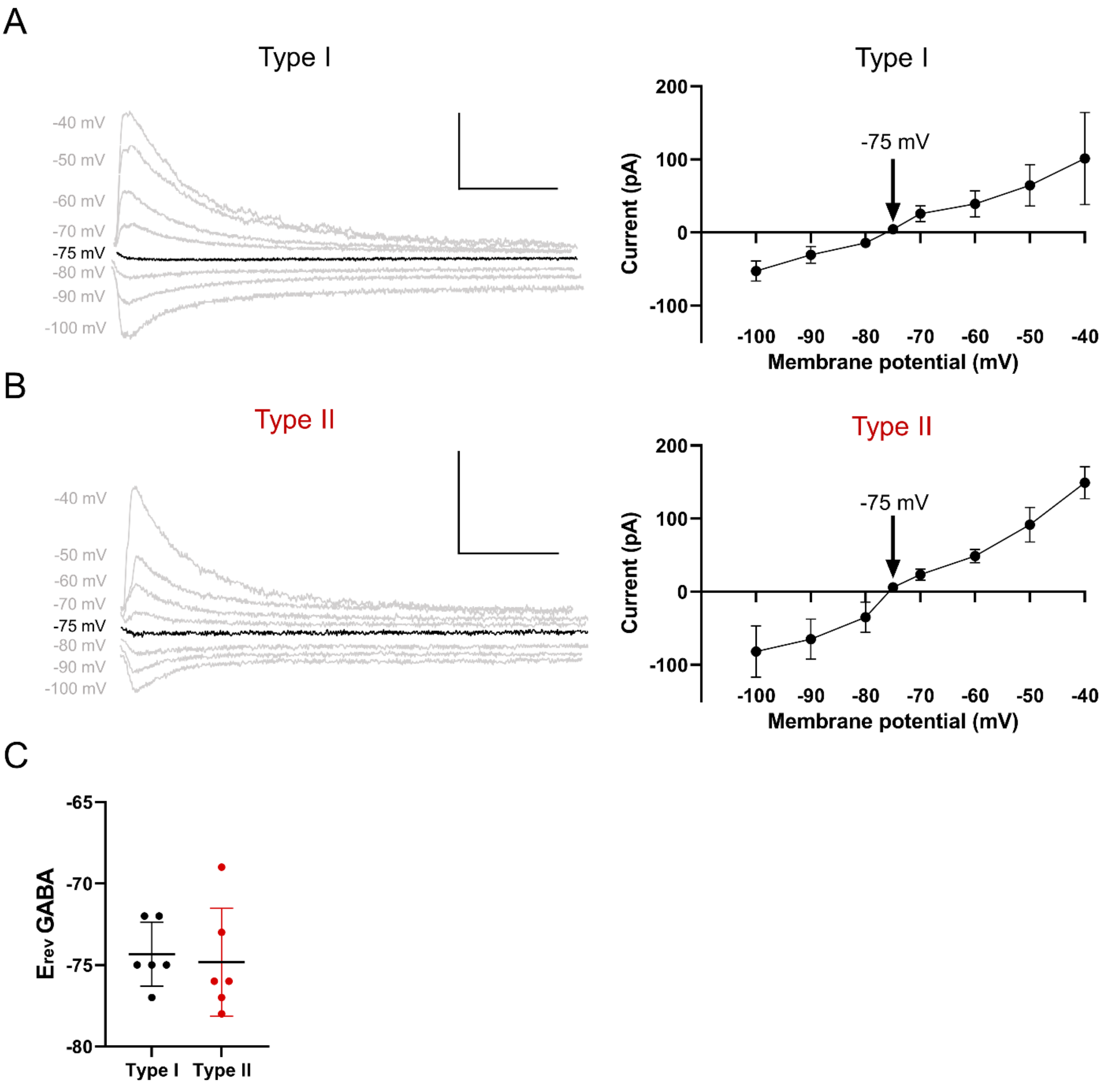

Figure S3. Reversal potential of GABA-mediated currents for both claustrum projection neuron subtypes. Related to STAR Methods section. A) Input/output

curve across multiple membrane potentials revealed a reversal potential of $GABA_A$ receptor-mediated currents to be approximately -75mV for type I and B) type II neurons. C) There was no significant difference between type I and II $GABA_A$ receptor-mediated current reversal potentials. n=6 type I cells; n=6 type II cells; n=12 cells total. Horizontal scale bars: A-B) 20ms. Vertical scale bars: A-B) 200pA. Error bars: standard error of the mean.

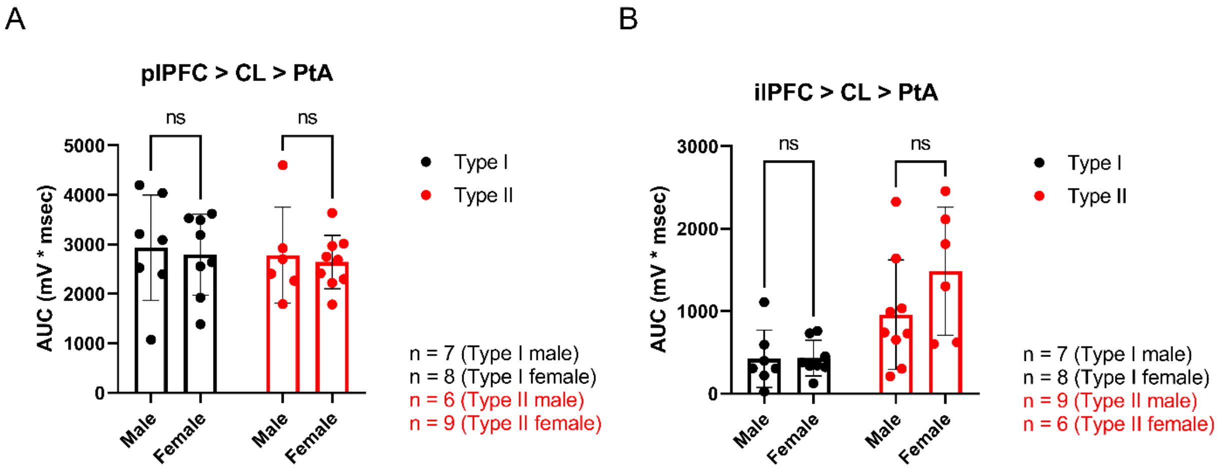

Figure S4. Area under the curve analyses reveal no sex differences in select cortico-claustro-cortical strength. Related to Figure 4. A representative strong circuit (plPFC > CL > PtA) (A) and relatively weak circuit (ilPFC > CL > PtA) (B) AUC values for both neuron subtypes are not statistically significant between male and females. A) n=7 type I cells (male), n=8 type I cells (female); n=6 type II cells (male), n=9 type II cells (female). B) n=7 type I cells (male), n=8 type I cells (female); n=9 type II cells (male), n=6 type II cells (female). A) Two-Way ANOVA with Sidak's Multiple

Comparisons test (P = 0.9370 Type I; 0.9412 Type II). B) Two-Way ANOVA with Sidak's Multiple Comparisons test (P = 0.9994 Type I; 0.1458 Type II).

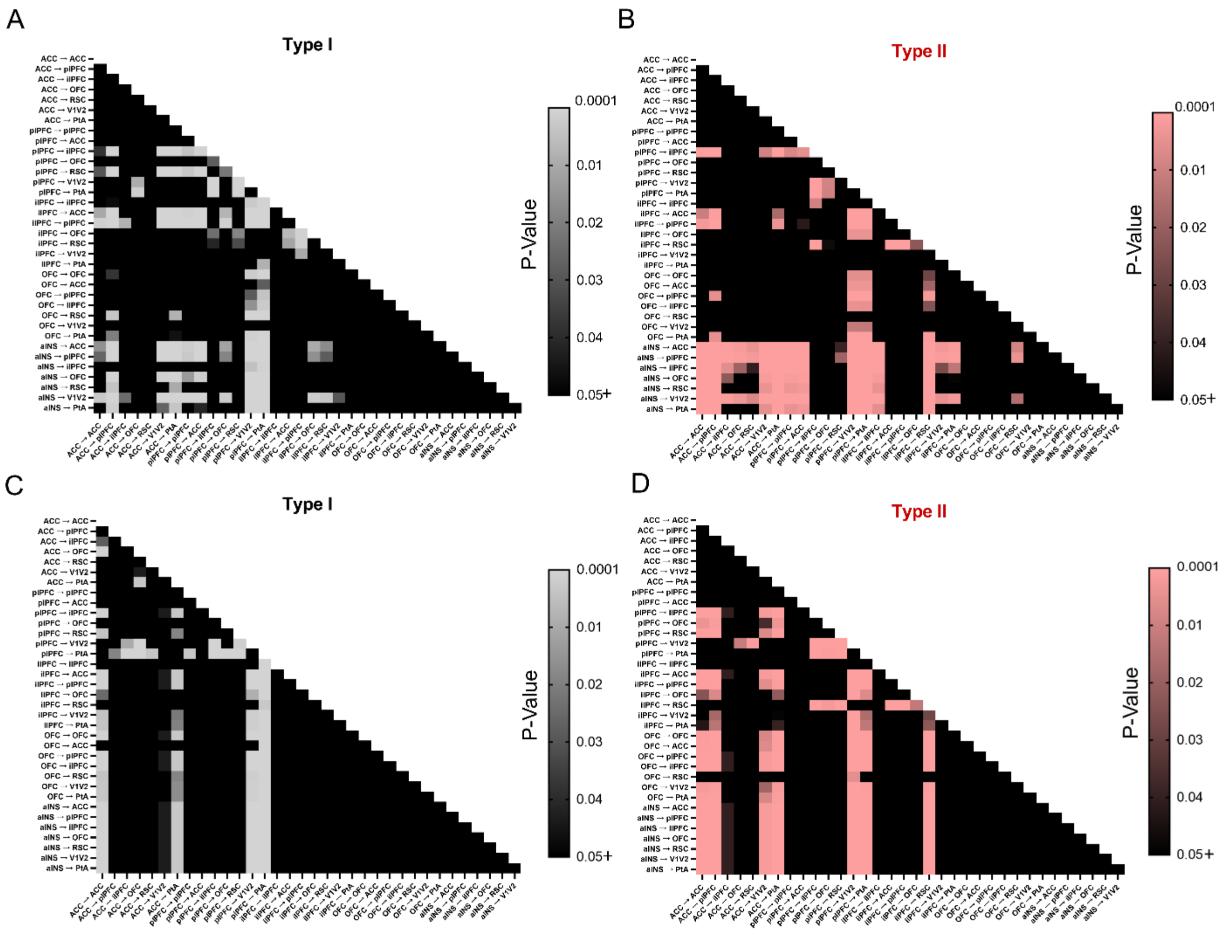

Figure S5. Multiple comparisons test for average area under the curve and action potentials per light pulse values revealed specificity in claustrum neuron activation depending on projection output target region. Related to Figures 4 and 5. A-B) The Kruskal Wallis test was used to generate a multiple comparisons P-value matrix for average AUC comparisons for type I (A) and type II (B) trans-claustral circuits. C-D) The Kruskal Wallis test was used to generate a multiple comparisons P-value

matrix for average action potentials per light pulse for type I (C) and type II (D) trans-claustral circuits.
